# Circular RNA hsa_circ_0001649 inhibits hepatocellular carcinoma progression *via* multiple miRNAs sponge

**DOI:** 10.18632/aging.101988

**Published:** 2019-05-28

**Authors:** Yang Su, Chao Xu, Yuting Liu, Yilin Hu, Haiyan Wu

**Affiliations:** 1 Department of Hepatobiliary and Pancreatic Surgery, The Affiliated Huaian No.1 People’s Hospital of Nanjing Medical University, Huaian, China; 2 State Key Laboratory of Reproductive Medicine, Center for Global Health, Key Laboratory of Modern Toxicology of Ministry of Education, School of Public Health, Nanjing Medical University, Nanjing, China; 3 Research Center of Clinical Medicine, Nantong University Affiliated Hospital, Nantong, China

**Keywords:** circRNA, proliferation, migration, hepatocellular carcinoma, ceRNA

## Abstract

Circular RNA (circRNA) exerts an essential role in tumor development. Hsa_circ_0001649 (circ-0001649) was produced at the *SHPRH* gene locus containing exon 26-29. This study analyzed the specific mechanism of circ-0001649 in influencing the development of hepatocellular carcinoma (HCC). Relative levels of circ-0001649 in HCC cell lines and tissues were examined by qRT-PCR. The direct binding between circ-0001649 and miR-127-5p/miR-612/miR-4688 were verified through Dual-luciferase reporter gene assay, RNA Binding Protein Immunoprecipitation (RIP) assay and western blot detection. *In vitro* and *in vivo* regulatory roles of circ-0001649 in proliferative and migratory abilities of HCC were evaluated by EdU, Transwell and tumourigenicity assay, respectively. Results showed that circ-0001649 was markedly decreased in hepatocellular carcinoma cell lines and tumor tissues. Overexpression of circ-0001649 greatly inhibited proliferation and migration of HCC *in vitro* and *in vivo*. More importantly, we confirmed that circ-0001649 regulated cellular behaviors of HCC cells by targeting *SHPRH*. Furthermore, we determined that circ-0001649 served as a ceRNA to sponge miR-127-5p, miR-612 and miR-4688, thus activating *SHPRH.* In summary, our study showed that circ-0001649 was lowly expressed in HCC and inhibited HCC progression *via* multiple miRNAs sponge.

## INTRODUCTION

Hepatocellular carcinoma (HCC) is a common malignancy, which is also a leading cause of tumor death throughout the world [1]. HCC is one of the malignant tumors with high morbidity and mortality. It is characterized with high malignancy, poor prognosis, strong invasion and metastasis [2]. Therefore, searching for molecular markers that are responsible for the occurrence and development of HCC is essential for inhibiting its malignant progression. Currently, relative genes in the pathogenesis of HCC have been identified, including *TP53, PTEN, BMI1* and etc [3–5]. Meanwhile, some non-coding RNAs are confirmed to be involved in the pathological progression of HCC, such as miRNA-448, lncRNA *FEZF1-AS1* and circRNA_104075 [6–8]. The exact pathogenesis of HCC still needs to be fully explored. Researches on the pathogenesis of HCC from the perspective of epigenetics are of great clinical value.

CircRNA, a kind of noncoding RNA, was firstly identified in 1976 [9]. These RNAs present a complete closed loop structure without poly-A tail. CircRNAs have not been well concerned previously because traditional RNA detection methods had multiple limitations. Recently, important roles of circRNAs in many physiological and pathological behaviors have been identified, such as regulation of proliferation, differentiation and metastasis of cancer cells [10]. Relative circRNAs in the pathogenesis of HCC have also been discovered. Han D et al suggested that circMTO1 suppresses HCC progression by sponging microRNA-9 [11]. Yao Z et al proved that both *ZKSCAN1* and its related circZKSCAN1 inhibit proliferative, migratory, and invasive potentials of HCC cells *via* different transduction pathways [12]. In-depth researches on circRNAs in the development of HCC contribute to significant clinical value.

Competing endogenous RNAs (ceRNAs) are transcripts that sponging target miRNAs, modulating post-transcriptional regulation *via* completely binding to the target miRNAs [13]. Nowadays, circRNAs gradually become the famous members in ceRNA family with the function of harboring a great number of conserved miRNA response elements (MREs) [14]. Some circRNAs are responsible for tumor initiation and progression as ceRNAs. Xie B pointed out the involvement of has_circ_0078710 in HCC progression that is dependent on sponging microRNA-31 [15]. Zhang X indicated that circRNA_104075 stimulates YAP-dependent tumorigenesis through regulating *HNF4a*, showing a diagnostic value in HCC [8]. Researches conducted by Zhang X and Qin M both confirmed the potential role of lowly expressed circ-0001649 as a biomarker for HCC. Study of Qin M showed that hsa_circ_0001649 expression was significantly downregulated in HCC tissues (p = 0.0014) based on an analysis of 89 paired samples of HCC and adjacent liver tissues and the area under the ROC curve (AUC) was 0.63 [16]. Meanwhile, hsa_circ_0001649 expression was measured in 77 pairs of HCC and adjacent no-tumor tissues by quantitative Real-Time polymerase chain reaction and Zhang X verified that hsa_circ_0001649 was down-regulated in HCC tissues compared with adjacent non-tumor tissues [17].

In this study, circ-0001649 was found to be lowly expressed in HCC cell lines and tumor tissues. Circ-0001649 overexpression markedly inhibited migratory and invasive capacities of HCC cells. Through dual-luciferase reporter gene assay, we confirmed the ceRNA effect of circ-0001649 on sponging miR-127-5p, miR-612 and miR-4688, thereafter mediating *SHPRH* expression. Finally, rescue experiments verified the promotive role of circ-0001649/miRNAs/*SHPRH* axis in HCC progression through regulating migratory and proliferative capacities.

## RESULTS

### Circ-0001649 characteristics in HCC

**Figure 1:**
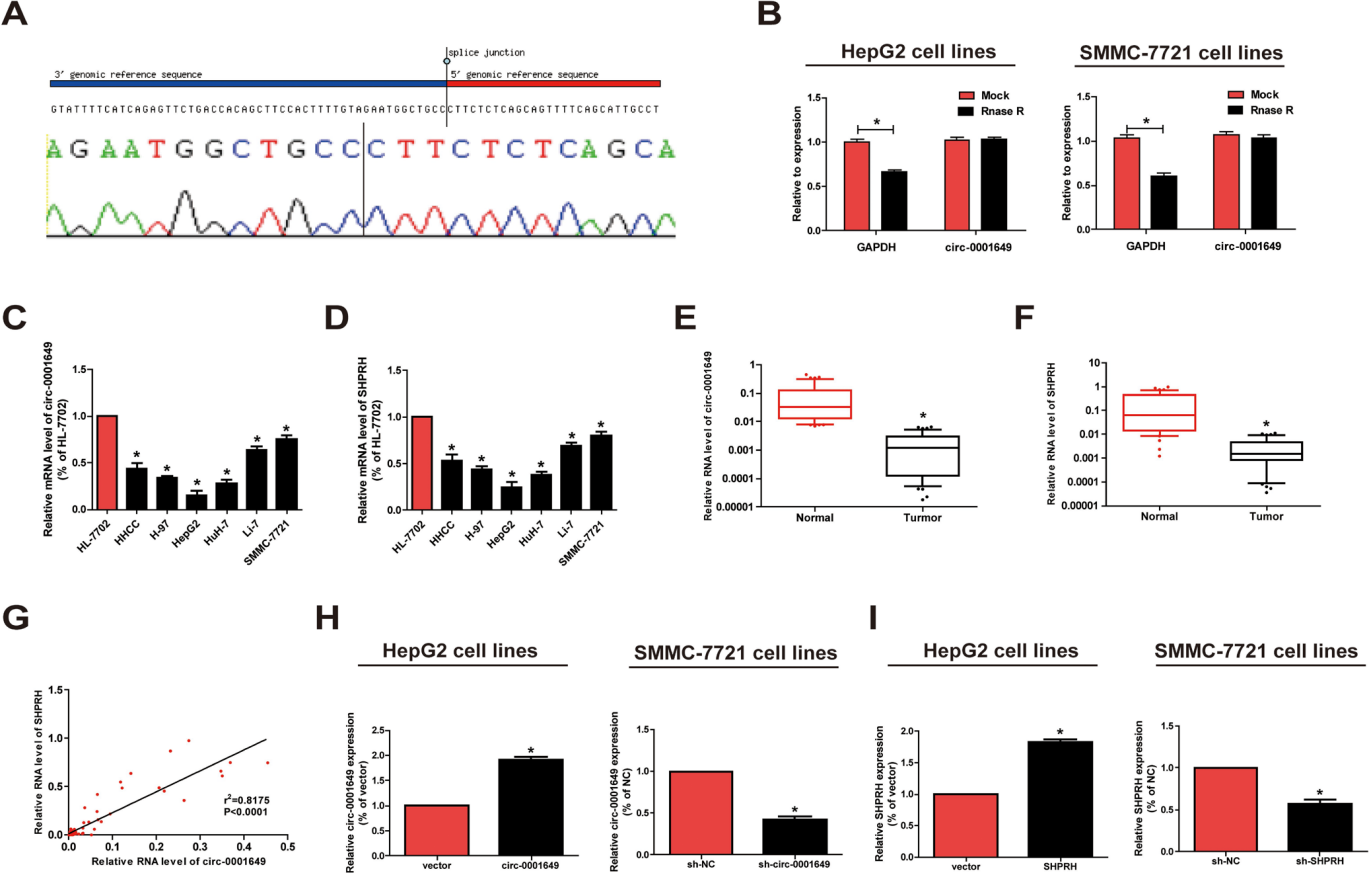
**Characteristics and expression of circ-0001649 in HCC.** (**A**) The sequence of circ-0001649 in circBase (upper panel) was consistent with the result of Sanger sequencing (lower panel). (**B**) Circ-0001649 was resistant to RNaseR digestion in HCC cell lines. (**C**) Circ-0001649 expression was verified by qRT-PCR in HHCC, H-97, HepG2, HuH-7, Li-7, SMMC-7721 cell lines and normal cell line HL-7702. Compared with HL-7702 cells, the expression of circ-0001649 in liver cancer cells was significantly reduced. Its expression was the lowest in HepG2 cells and the highest in SMMC-7721 cells. (**D**) *SHPRH* expression was verified by qRT-PCR in HCC cell lines and normal cell line HL-7702. (**E**) qRT-PCR detection of the relative expression of circ-0001649 in paired HCC tumor and normal tissues (n=84). (**F**) qRT-PCR detection of the relative expression of *SHPRH* in paired HCC tumor and normal tissues (n=84). (**G**) Correlation between circ-0001649 and *SHPRH* in HCC samples. (**H**) Expression of circ-0001649 was upregulated in circ-0001649 overexpression group and downregulated in the sh-circ-0001649 group. (**I**) Expression of *SHPRH* was upregulated in the *SHPRH* overexpression group and downregulated in the sh-*SHPRH* group. *P<0.05, data represent the mean ± SD.

Firstly, we confirmed that the circ-0001649 sequence amplified by the primer was identical to its sequence in Circbase by sanger sequencing (Figure 1A). To further verify the circular characteristics of circ-0001649, total RNA was treated with RNaseR to distinguish linear RNAs from circRNAs. We found that circ-0001649 was indeed circRNA, which was resistant to RNaseR digestion (Figure 1B). qRT-PCR was conducted to determine circ-0001649 and *SHPRH* in HCC cells and normal liver cells HL-7702. Circ-0001649 was lowly expressed in HCC cells than HL-7702 cells. In particular, SMMC-7721 and HepG2 cells presented a pronounced difference in circ-0001649 expression, and were utilized for next assays (Figure 1C, 1D). Furthermore, the results of circ-0001649 and *SHPRH* levels in HCC tumors and paired adjacent nonmalignant tissues showed significantly lower circ-0001649 and *SHPRH* in HCC tumor tissues (Figure 1E, 1F). Interestingly, there is a positive correlation between circ-0001649 and *SHPRH* in HCC samples (Figure 1G). Expression of circ-0001649 in 84 HCC cancer tissues was detected by qRT-PCR. HCC tumor tissues were divided into high circ-0001649 group (n=42) and low circ-0001649 group (n=42) according to the median value. Then we found that low expression of circ-0001649 in the tumor tissues was associated with grade of differentiation and tumor satellite, but not with other clinicopathological features including gender, age, tumor diameter, serum α-fetoprotein (AFP), liver function (Child-Pugh stage), hepatocirrhosis, HBV infection or HCV infection (Supplementary Table 1). As shown in Supplementary Figure 1A, the expression level of circ-0001649 in the stable HBV-producing cell line HepG2.2.15 was similar to that in its parental cell line HepG2. In addition, circ-0001649 expression was similar in HCC cells transfected with pHBV1.3 copy (the HBV1.3 expression plasmid) compared to the control (Supplementary Figure 1B). These results suggest that there is no significant correlation between circ-0001649 and HBV, consistent with the clinicopathological characteristics. To further clarify the potential function of circ-0001649, circ-0001649 shRNA and circ-0001649 overexpression vector were constructed. QRT-PCR verified their sufficient transfection efficacy (Figure 1H). Similarly, transfection efficacy of *SHPRH* shRNA and *SHPRH* overexpression vector were tested as well (Figure 1I).

### Circ-0001649 and *SHPRH* regulated proliferative and migratory abilities of HCC cells

**Figure 2:**
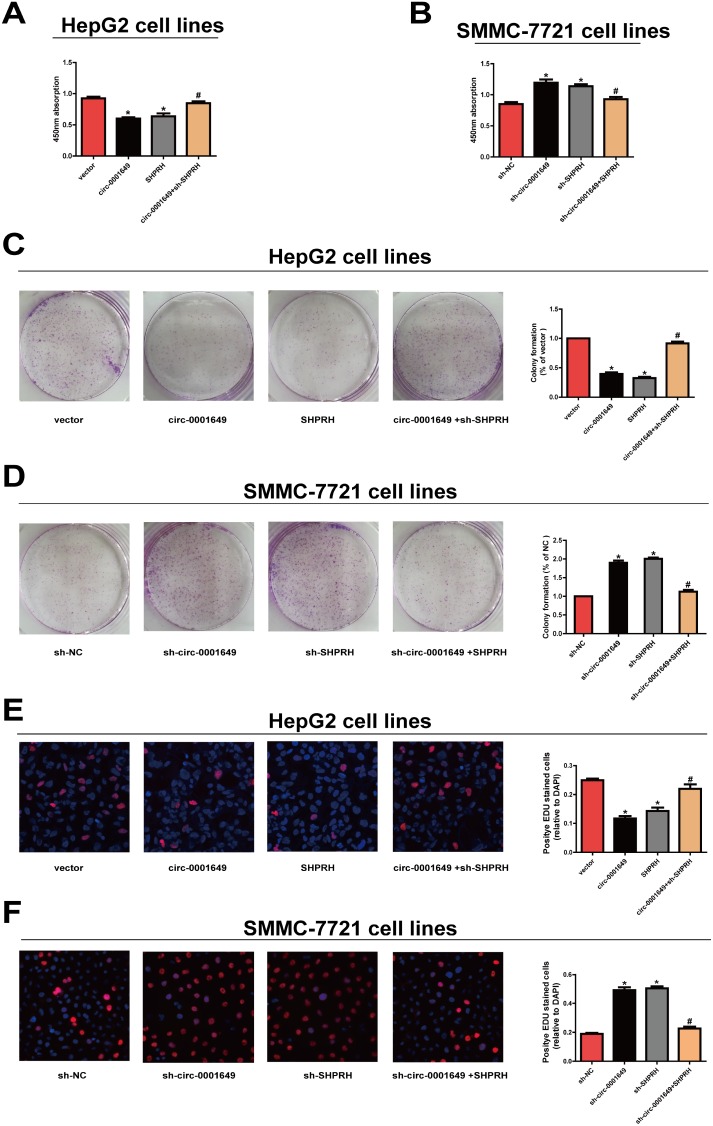
**Circ-0001649 and *SHPRH* regulated proliferative ability of HCC cells.** (**A**), (**C**), (**E**) CCK-8 assay, colony formation assay and EdU assay showed that upregulation of circ-0001649 inhibited the proliferation of HCC cells, which was reversed by sh-*SHPRH*. (**B**), (**D**), (**F**) CCK-8 assay, colony formation assay and EdU assay showed that downregulation of circ-0001649 promoted the proliferation of HCC cells, which was reversed by *SHPRH*. *P < 0.05 versus control group, ^#^ *P* < 0.05 versus circ-0001649 or sh-circ-0001649 group, data represent the mean ± SD.

Proliferative capacity in HCC cells was evaluated using CCK-8, colony formation and EdU assay. Overexpression of circ-0001649 or *SHPRH* remarkably reduced proliferative rate, while circ-0001649 or *SHPRH* knockdown obtained the opposite result (Figure 2A, 2B). Moreover, colony formation (Figure 2C, 2D) and EdU assay (Figure 2E, 2F) yielded the identical results as CCK-8 assay indicated.

**Figure 3:**
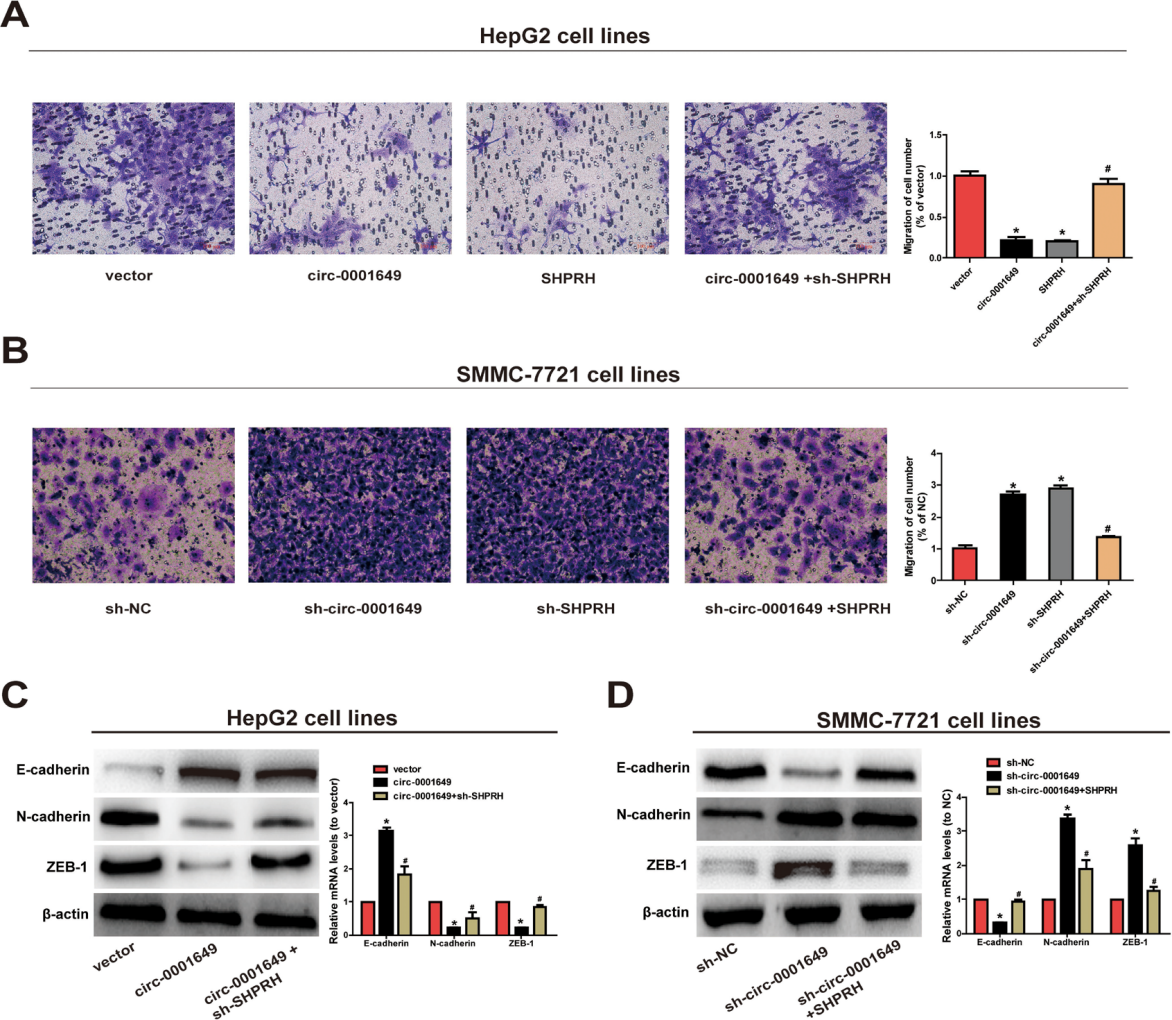
**Circ-0001649 and *SHPRH* regulated migration of HCC cells.** (**A**) Transwell assay showed that upregulation of circ-0001649 inhibited cell migration, which was reversed by sh-*SHPRH*. (**B**) Conversely, downregulation of circ-0001649 promoted cell migration, which was reversed by *SHPRH*. (**C**) The protein and mRNA levels of epithelial marker, mesenchymal marker and EMT-related transcriptional active factor in HepG2 cells upon transfection of circ-0001649 overexpression vector or co-transfection of circ-0001649 overexpression vector and sh-*SHPRH*. (**D**) The protein and mRNA levels of epithelial marker, mesenchymal marker and EMT-related transcriptional active factor in SMMC-7721 cells upon transfection of sh-circ-0001649 or co-transfection of sh-circ-0001649 and *SHPRH* overexpression vector. *P < 0.05 versus control group, ^#^ *P* < 0.05 versus circ-0001649 or sh-circ-0001649 group, data represent the mean ± SD.

Next, Transwell assay was conducted to evaluate the abilities to migrate. Migratory capacity of HCC cells was downregulated in cells overexpressing circ-0001649 and *SHPRH*, while the opposite result was observed after knockdown of circ-0001649 or *SHPRH* (Figure 3A, 3B). As EMT has a vital role in HCC cell migration, we further explored the effect of circ-0001649/*SHPRH* on epithelial features. The expression of epithelial marker *E-cadherin*, and mesenchymal marker *N-cadherin* were detected. As shown in Figure 3C, ectopic expression of circ-0001649 significantly induced the expression of *E-cadherin*, conversely reduced *N-cadherin* expression at protein and mRNA levels. Consistently, the expression of EMT-related transcription factor *ZEB-1* was downregulated upon overexpression of circ-0001649. Meanwhile, down-regulation of *SHPRH* reversed these changes (Figure 3C). As shown in Figure 3D, down-regulation of circ-0001649 significantly induced the expression of *N-cadherin*, conversely reduced *E-cadherin* expression at protein and mRNA levels. Consistently, the expression of EMT-related transcription factor *ZEB-1* was upregulated upon inhibition of circ-0001649. Meanwhile, up-regulation of *SHPRH* reversed these changes (Figure 3D). These data suggested that circ-0001649 and *SHPRH* were capable of inhibiting abilities of HCC cells to proliferate and migrate.

### Circ-0001649 inhibited *in vivo* proliferative capacity of HCC cells

**Figure 4:**
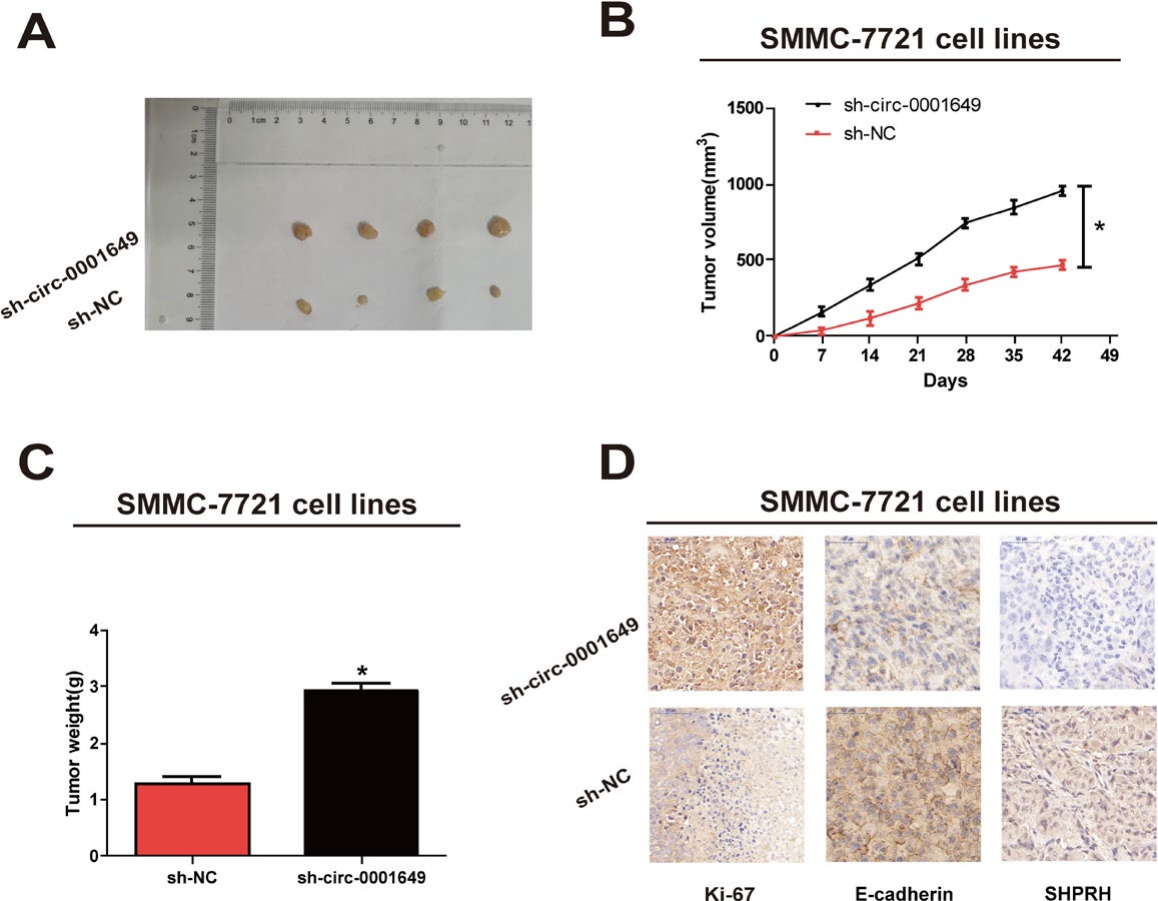
**Circ-0001649 regulated *in vivo* proliferation of SMMC-7721 cells.** (**A**) Representative images of xenografts tumor (four mice per group) in nude mice. (**B**) Tumor growth curves were measured after injection of SMMC-7721 cells transfected with sh-circ-0001649 or sh-NC. (**C**) Tumor weights were measured. (**D**) *Ki-67, SHPRH* and *E-cadherin* IHC staining. *P<0.05, data represent the mean ± SD.

*In vivo* proliferative capacity of circ-0001649 was analyzed in nude mice. To verify whether circ-0001649 could affect HCC tumorigenesis, SMMC-7721 cells were transfected with sh-NC or sh-circ-0001649. After 6 weeks, tumor growth was pronounced (Figure 4A), and tumor weight and volume were strikingly enhanced in the sh-circ-0001649 group (Figure 4B, 4C). Consistently, immunostaining of xenografted tumor tissues showed upregulated expression of *Ki-67* and downregulated expression of *SHPRH* and *E-cadherin* in the sh-circ-0001649 group (Figure 4D). These data suggested that circ-0001649 was involved in HCC tumorigenesis and inhibited *in vivo* proliferative capacity of HCC cells.

### Circ-0001649 exerted its functions through sponging miR-127-5p, miR-612 and miR-4688

**Figure 5:**
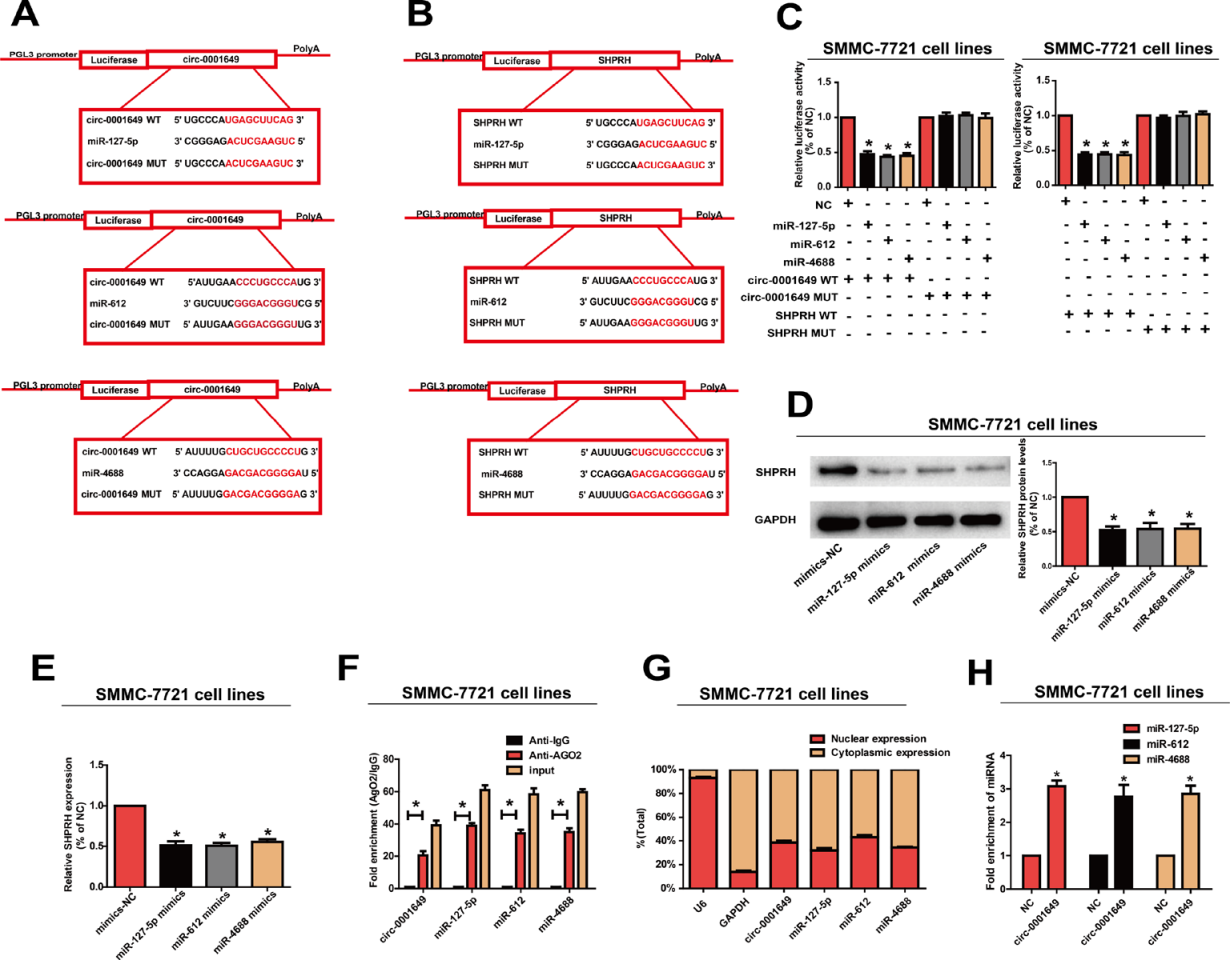
**Circ-0001649 served as a sponge for miR-127-5p, miR-612 and miR-4688 to regulated SHPRH.** (**A**, **B**) Circ-0001649 and SHPRH were the potential targets for miR-127-5p, miR-612 and miR-4688. (**C**) Luciferase activity in SMMC-7721 cells after transfection with negative control or miRNAs mimics. (**D**, **E**) Overexpression of miR-127-5p, miR-612 or miR-4688 inhibited protein and mRNA levels of SHPRH. (**F**) RIP assay confirmed the binding relationships. (**G**) MiR-127-5p, miR-612, miR-4688 and circ-0001649 were mainly distributed in cytoplasm. (**H**) Compared with the negative control, miR-127-5p, miR-612 and miR-4688 were pulled down and detected in the circ-0001649 biotinylated probed RNA-RNA complexes by qRT-PCR. *P<0.05, data represent the mean ± SD.

By searching RegRNA, Starbase, miRDB and Targetscan, we found that both circ-0001649 and *SHPRH* were potential targets of miR-127-5p, miR-612 and miR-4688 (Figure 5A, 5B). Next, dual-luciferase reporter assay confirmed the online search. Luciferase activity decreased in SMMC-7721 cells co-transfected with the searched miRNA mimic and wild-type of target gene. However, mutant-type group did not show obvious changes in luciferase activity (Figure 5C). Subsequently, Western blot analysis revealed that mimics of miR-127-5p, miR-612 or miR-4688 in SMMC-7721 cells downregulated protein expression of *SHPRH* (Figure 5D). QRT-PCR yielded the same results at the mRNA level (Figure 5E).

MiRNA distributes in the cytoplasm, which is a component of the RNA-induced silencing complex (RISC) containing Ago2. Ago2 is required for miRNA-mediated gene silencing. In this study, we analyzed if circ-0001649 and miR-127-5p, miR-612 or miR-4688 contained the same RISC and performed RIP assay in SMMC-7721 cells. It is shown that circ-0001649 was enriched in Ago2-containing miRNAs than IgG control. MiR-127-5p, miR-612 and miR-4688 were also detected in the precipitate (Figure 5F). To identify the subcellular localization of circ-0001649, miR-127-5p, miR-612 and miR-4688, nuclear and cytoplasmic fractions were extracted, respectively. U6 and GAPDH were utilized as the nuclear and cytoplasmic internal reference, respectively. Our results revealed that circ-0001649, miR-127-5p, miR-612 and miR-4688 were mainly distributed in the cytoplasm of SMMC-7721 cells, suggesting their post-transcriptional regulatory potentials (Figure 5G). Pull-down assays with a circ-0001649 biotinylated probe were then performed. MiR-127-5p, miR-612 and miR-4688 were pulled down and detected in the circ-0001649 biotinylated probed RNA-RNA complexes by qRT-PCR (Figure 5H). These results suggested that circ-0001649 regulated HCC cells by targeting *SHPRH* as a ceRNA.

### Circ-0001649/miRNAs axis was critical for cell function

**Figure 6:**
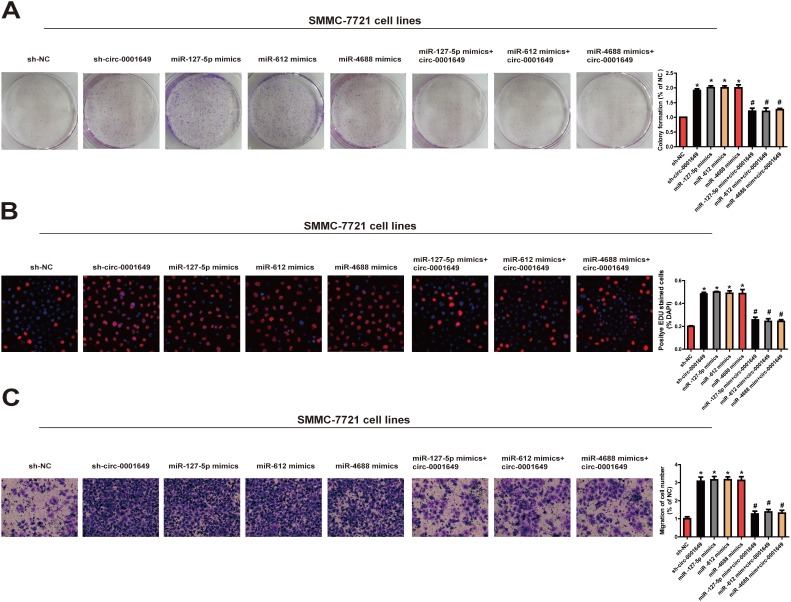
**Circ-0001649/miRNAs axis was critical for cell function.** Colony formation assay (**A**), EdU assay (**B**) and Transwell migration assay (**C**) were performed to determine the proliferation and migration in cells transfected with miRNAs mimics and circ-0001649 overexpression vector. *P < 0.05 versus control group, ^#^ *P* < 0.05 versus corresponding miRNA mimics group, data represent the mean ± SD.

Biological functions of miR-127-5p, miR-612 and miR-4688 in regulating behaviors of HCC cells were then clarified. The results showed that the proliferative (Figure 6A, 6B) and migratory potentials (Figure 6C) of SMMC-7721 cells were up-regulated in the sh-circ-0001649, miR-127-5p mimics, miR-612 mimics and miR-4688 mimics groups. These data indicated that the effect of circ-0001649 in HCC was partially relying on miR-127-5p, miR-612 and miR-4688.

**Figure 7:**
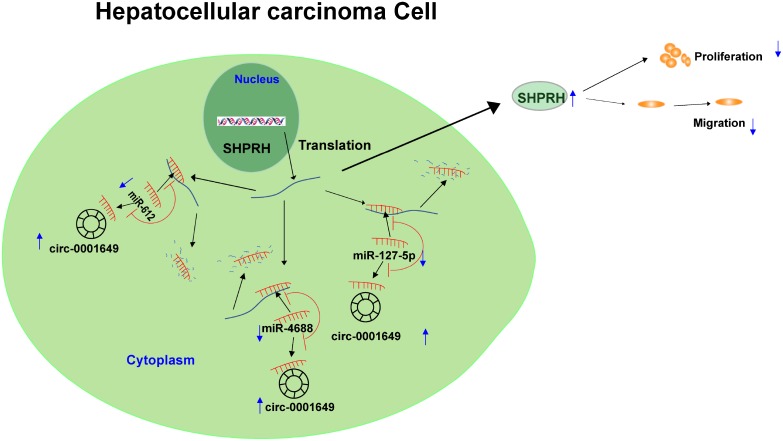
**Graphical abstract of how circ-0001649 inhibits hepatocellular carcinoma progression.** A schematic model of circ-0001649/miRNAs/SHPRH signaling pathway in hepatocellular carcinoma. circ-0001649 competitively binds to miR-127-5p, miR-612 and miR-4688, resulting in upregulation of SHPRH. Furthermore, upregulation of SHPRH inhibits the clonogenicity, migration and proliferation of HCC cell lines.

To sum up, circ-0001649 competitively bound to miR-127-5p, miR-612 and miR-4688 to upregulate *SHPRH* level, thereafter inhibiting clonogenic, proliferative and migratory abilities of HCC cells (Figure 7).

## DISCUSSION

Recently, accumulating studies have pointed out that circRNAs are involved in the pathogenesis of HCC, and serve as diagnostic and therapeutic targets for HCC [18]. CircRNAs relative to HCC are differentially expressed [19, 20]. Our study found that circ-0001649 was lowly expressed in HCC cell lines and tumor tissues, which were consistent with previous studies [16, 17]. Subsequently, *in vivo* and *in vitro* experiments all indicated that circ-0001649 knockdown accelerated proliferative and migratory potentials of HCC cells and activated EMT signaling pathway. Our findings showed the crucial role of circ-0001649 in the progression of HCC.

MiRNAs are encoded by endogenous genes, which is a class of non-coding, single-stranded RNAs with 22 nucleotides in length. Functionally, they mediate gene expressions in plants and animals at post-transcriptional level [21]. So far, 28,645 miRNAs have been found in animals, plants and viruses [22]. Most miRNAs exist in the genome in the form of single copies, multiple copies, or gene clusters [23]. Some miRNAs have been identified to participate in the tumorigenesis of HCC through downregulating the target genes [24]. Previous studies limited on a certain miRNA that exerts biological function in the circRNA network [25]. Here, we first identified all relative miRNAs to circ-0001649 and *SHPRH* through online prediction, that were miR-127-5p, miR-612 and miR-4688. Studies have clarified that miR-127-5p [26, 27] and miR-612 [28–30] participate in the pathogenesis of HCC. However, miR-4688 in HCC development is rarely reported. CircRNAs serve as miRNA sponges, reversing the inhibitory effect of miRNA on target gene expressions [31]. Our functional experiments proved the interaction among circ-0001649, miRNAs and its host gene *SHPRH* in HCC cells.

SNF2 histone linker PHD RING helicase (*SHPRH*) locates in 6q24.3, which is the host gene of circ-0001649. *SHPRH* is ubiquitously expressed, containing motifs of some DNA repair proteins, transcriptional factors, and helicases. It is a functional homolog of S. cerevisiae RAD5 [32]. *SHPRH* coordinates post-replication repair and prevent mutations with different formations relying on a lesion-specific manner [33]. Such characteristics of *SHPRH* indicate that downregulation of *SHPRH* may be important in mutagenesis of oncogenes. In this study, *SHPRH* was positively associated with circ-0001649 and lowly expressed in HCC cell lines and tumor tissues. Interestingly, *SHPRH* expression was inhibited in HCC cells transfected with miRNAs mimics. We concluded that circ-0001649 and its host gene *SHPRH* were downregulated in HCC, and circ-0001649 regulated the expression of *SHPRH* through sponging miRNAs as a ceRNA. It is noteworthy that our research revealed more than one miRNA that were competitively bound to circ-0001649. It may provide novel directions in other disease explorations.

## MATERIALS AND METHODS

### Study subjects and design

All the subjects singed the written informed and the study protocol received the approval from the Ethics Committee of Nantong University Affiliated Hospital. This study analyzed 84 matched tumor and paired adjacent nonmalignant tissues from HCC patients of Nantong University Affiliated Hospital.

### Cell culture and transfection

HHCC, H-97, HepG2, HuH-7, Li-7, SMMC-7721, HepG2.2.15 and HL-7702 cell lines were purchased from the Shanghai Cell Bank of Chinese Academy of Sciences (Shanghai, China). Cells were maintained in Dulbecco’s Modified Eagle medium (DMEM) (Hyclone, UT, USA) with 10% fetal bovine serum (FBS, Beyotime, Nantong, China), 100 μg/ml streptomycin and 100 IU/ml penicillin (Invitrogen, USA) at 37°C, 5% CO_2_. For transfections, cells at the confluence of 50–80% were infected with 1 × 10^6^ recombinant lentivirus-transducing units and 6 μg/mL Polybrene (Sigma, Shanghai, China). Stably transfected cells were selected via treatment with 2μg/mL puromycin for 2 weeks. Stably transfected cells were picked via flow cytometry for subsequent assays. Plasmid, lentivirus, miRNA inhibitor and miRNA mimics used in this study were purchased from GenePharma Co., Ltd. (Shanghai, China), pHBV1.3 copy was purchased from Miaolingbio (Wuhan, China). Lipofectamine 3000 (Invitrogen, CA, USA) was utilized for transfection.

### RNA isolation and qRT-PCR

Total RNA was extracted from tissues or cells using TRIzol reagent (Life Technologies, CA, USA) and quantified by NanoDrop 2000 Spectrophotometer (Thermo Scientific, Wilmington, DE, USA). Those qualified RNAs were reversely transcribed using the Reverse Transcription Kit (Takara, Tokyo, Japan). QRT-PCR was conducted (SYBR Premix Ex Taq, Takara, Tokyo, Japan) on Light Cycler 480 (Roche, Switzerland). Relative expressions of circ-0001649, miRNAs and *SHPRH* were finally calculated.

### RNase R digestion

Total RNA (5 μg) was cultured with 3U/μg of RNase R (Epicentre Biotechnologies, Shanghai, China) for 15 min at 37°C. The RNase R digestion was performed twice as previously described.

### Sanger sequence

The amplified product was inserted into the T vector for Sanger sequencing. After determination of the full length, different primers were constructed to verify the back-splice joint of circ-0001649: 5′-AATGCTGAAAACTGCTGAGAGAA-3′ (sense) and 5′-TTGAGAAAACGAGTGCTTTGG-3′ (antisense). We authorized Invitrogen (Shanghai, China) to construct the primers, and Realgene (Nanjing, China) to perform Sanger sequencing.

### Cell proliferation assay

Cells in 96-well plates were cultured with CCK-8 (Beyotime, Nantong, China). Absorbance at 450 nm was recorded by the TECAN infinite M200 Multimode microplate reader (Tecan, Mechelen, Belgium). Proliferation was also determined by 5-ethynyl-2'-deoxyuridine (EdU) assay. Procedures were independently repeated in triplicate.

### Colony formation assay

1×10^3^ cells were seeded per well in 6-well plates. Cells were maintained for 1 week in medium containing 10% FBS. Colonies containing over 30 cells in each well were captured and counted.

### Cell migration assay

100 μL cell suspension was seeded in the upper chamber with serum-free medium, and 600 μL medium with 10% FBS was supplied in the bottom chamber. 24 h later, cells were dyed with crystal violet (Beyotime, Nantong, China), counted and captured with five fields per well (magnification 40×). Procedures were independently repeated in triplicate.

### Dual-luciferase reporter gene assay

Wild-type plasmids circ-0001649-WT and *SHPRH*-WT, as well as mutant-type plasmids circ-0001649-MUT and *SHPRH*-MUT were inserted into the pGL3 promoter vector (GenePharma, Shanghai, China). SMMC-7721 cells seeded into 24-well plate were co-transfected 50 nM miRNAs mimics or negative control and 80 ng plasmid with 5 ng pRL-SV40 using Lipofectamine 3000. Luciferase activity was recorded using the dual-luciferase reporter assay kit (Promega, Madison, WI, USA).

### Chromatin fractionation

Cytoplasmic and nuclear RNA were extracted with the PARIS Kit (Life Technologies, USA). QRT-RCR was performed to determine cytoplasm and nuclear RNA with the internal references of GAPDH and U6, respectively.

### RNA binding protein immunoprecipitation (RIP) assay

Magna RIP RNA-Binding Protein Immunoprecipitation Kit (Millipore, Billerica, MA, USA) was used for RIP assay. SMMC-7721 cells were lysed in RIP lysis buffer, and RNAs magnetic beads were conjugated to anti-AGO2 (ab32381, Abcam, Cambridge, MA, USA) or isotype-matched control anti-IgG (Millipore, Billerica, MA, USA). Relative expressions of circ-0001649 and miRNAs were finally quantified by qRT-PCR.

### RNA pull-down assay

SMMC-7721 cells transfected with biotinylated circ-0001649 or negative control (GenePharma, Shanghai, China) were lysed, incubated with Dynabeads M-280 Streptavidin (Invitrogen) and probe-coated beads at 4°C overnight. After elution of binding RNA complexes on these beads, they were quantified by qRT-PCR.

### Immunohistochemical staining

Briefly, the subcutaneous tumor was resected and fixed in 10% formaldehyde. After incubation with anti-*Ki-67* antibodies (1:200, Abcam, Cambridge, UK) , anti-*SHPRH* (1:100, Abcam, Cambridge, UK) and anti-*E-cadherin* (1:200, Abcam, Cambridge, UK) overnight, we visualize the slide using streptavidin-horseradish peroxidase-conjugated secondary antibody (Thermo Fisher Scientific, Waltham, MA, USA). Then Amplification and visualization were performed using DAB (Santa Cruz Biotechnology, Santa Cruz, CA, USA) substrate chromogen solution, followed by counterstaining with hematoxylin.

### Western blot

Proteins were isolated from cells by Radio Immunoprecipitation Assay (RIPA Beyotime, Nantong, China) buffer, electrophoresed on 10% sodium dodecyl sulphate-polyacrylamide gel electrophoresis (SDS-PAGE) and transferred to PVDF membranes (Millipore, Billerica, MA, USA). After 1 hour blockage in 5% skim milk, membranes were incubated with anti-*GAPDH* (Beyotime, Nantong, China), anti-*SHPRH,* anti*-E-cadherin,* anti*-N-cadherin,* anti*-ZEB1 and* anti*-β-actin* (Abcam, Cambridge, MA, USA) at 4^o^C overnight. At the other day, blots were incubated with secondary anti-body (Beyotime, Nantong, China) for 1 h. Band visualization was conducted using the enhanced chemiluminescence reagent kit (Millipore, Billerica, MA, USA).

### Tumourigenicity assay

Animal procedures were in accordance to the guidelines of the responsible governmental animal ethics committee. 5-week-old nude mice were purchased from Shanghai Institute for Biological Sciences (SIBS) and housed in laminar airflow cabinets with pathogen-free condition. Mice were subcutaneously injected into the back with 1 × 10^6^ SMMC-7721 cells stably transfected with sh-circ-0001649 or sh-NC suspended in 100 μL Hank’s balanced salt solution. Tumor volume was observed once a week and calculated: V = (length × width^2^)/2. Tumors were harvested after mice were sacrificed at 6 weeks.

### Statistical analysis

Statistical analysis was carried out using the SPSS 22.0 software (SPSS Inc., Chicago, IL, USA). The paired t-test was performed to detect the differential expression of circ-0001649 and *SHPRH* in cancer tissues compared with adjacent nonmalignant tissues. The clinicopathological characteristics were evaluated using chi-square test or Fisher's exact test. Two group comparison and correlation analyses were calculated, respectively, with two-tailed Student’s t-test and linear regression test using GraphPad Prism 5 software (GraphPad Software Inc., La Jolla, CA, USA). Data were presented as mean ±SEM. *P < 0.05 was considered with significance.

## Supplementary Material

Supplementary Table and Figure
