# Supplementary material for: Circular RNA hsa_circ_0001649 inhibits hepatocellular carcinoma progression via multiple miRNAs sponge
Source: Aging (Albany NY). 2019 May 28;11(10):3362–75. doi: 10.18632/aging.101988 (PMC6813922; doi:10.18632/aging.101988)
Supplement: Supplementary Table and Figure [file aging-11-101988-s001.pdf]

SUPPLEMENTARY MATERIAL

Supplementary Table 1. Relationship between circ-0001649 expression and clinicopathological characteristics of HCC patients.

Clinicopathological characteristics	n	High expression	Low expression	χ^2	*P* value
**Total**	**84**	**42**	**42**		
**Gender**				0.263	0.608
Male	64	31	33		
Female	20	11	9		
**Age (years)**				0.048	0.823
≤54	41	21	20		
>54	43	21	22		
**Grade of differentiation**				7.336	**0.026***
Low	32	10	22		
Middle	30	18	12		
High	22	14	8		
**Tumor diameter (cm)**				0.191	0.662
≤5	40	21	19		
>5	44	21	23		
**Liver function (Child-Pugh stage)**				0.081	0.776
A	69	35	34		
B or C	15	7	8		
**Hepatocirrhosis**				0.933	0.334
Absent	24	14	10		
Present	60	28	32		
**HBV infection**				0.808	0.369
Absent	32	18	14		
Present	52	24	28		
**HCV infection**					1.000
Absent	83	42	41		
Present	1	0	1		
**AFP (ng/ml)**				0.763	0.382
≤20	44	24	20		
>20	40	18	22		
**Tumor satellite**				7.244	**0.007***
Absent	61	36	25		
Present	23	6	17		

Two-sided χ2 for all variables except HCV infection between Low expression group and High expression group, HCV infection was evaluated using Fisher's exact test, *P<0.05.

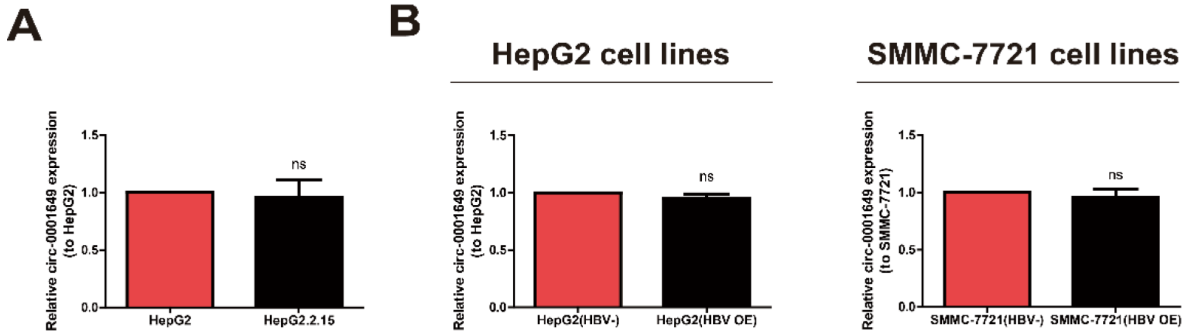

Supplementary Figure 1. Differential expression of circ-0001649 in HBV-related HCC cells. pHBV1.3 copy (the HBV1.3 expression plasmid) transfected into HepG2/SMMC-7721 cells, namely HepG2 (HBV OE) and SMMC-7721 (HBV OE), respectively. (**A**) The expression level of circ-0001649 in the stable HBV-producing cell line HepG2.2.15 was similar to that in its parental cell line HepG2. (**B**) After transfected with pHBV1.3 copy (the HBV1.3 expression plasmid), mRNA levels of circ-0001649 were detected by qRT-PCR. ns: no significant difference, data represent the mean ± SD.
